# Assessment of the paraspinal muscles of subjects presenting an idiopathic scoliosis: an EMG pilot study

**DOI:** 10.1186/1471-2474-6-14

**Published:** 2005-03-10

**Authors:** Nathaly Gaudreault, A Bertrand Arsenault, Christian Larivière, Sophie J DeSerres, Charles-Hilaire Rivard

**Affiliations:** 1 CRIR, Montreal Rehabilitation Institute, Montreal, Quebec, H3S 2J4, Canada; 2 School of Rehabilitation, Faculty of Medicine, University of Montreal, Montreal, Quebec, H3C 3J7, Canada; 3 Institut de recherche Robert-Sauvé en santé et en sécurité du travail (IRSST), Montreal, Quebec, H3A 3C2, Canada,. Reseach Center, Montreal Rehabilitation Institute, Montreal, Quebec, H3S 2J4, Canada; 4 School of physical and Occupational therapy, McGill Unversity, Montreal, Quebec, H3G 1Y5, Canada; 5 Department of surgery, Ste-Justine Hospital, Montreal, Quebec, H3A 3C2, Canada

**Keywords:** EMG, scoliosis, neuromuscular efficiency, muscle fatigue

## Abstract

**Background:**

It is known that the back muscles of scoliotic subjects present abnormalities in their fiber type composition. Some researchers have hypothesized that abnormal fiber composition can lead to paraspinal muscle dysfunction such as poor neuromuscular efficiency and muscle fatigue. EMG parameters were used to evaluate these impairments. The purpose of the present study was to examine the clinical potential of different EMG parameters such as amplitude (RMS) and median frequency (MF) of the power spectrum in order to assess the back muscles of patients presenting idiopathic scoliosis in terms of their neuromuscular efficiency and their muscular fatigue.

**Methods:**

L5/S1 moments during isometric efforts in extension were measured in six subjects with idiopathic scoliosis and ten healthy controls. The subjects performed three 7 s ramp contractions ranging from 0 to 100% maximum voluntary contraction (MVC) and one 30 s sustained contraction at 75% MVC. Surface EMG activity was recorded bilaterally from the paraspinal muscles at L5, L3, L1 and T10. The slope of the EMG RMS/force (neuromuscular efficiency) and MF/force (muscle composition) relationships were computed during the ramp contractions while the slope of the EMG RMS/time and MF/time relationships (muscle fatigue) were computed during the sustained contraction. Comparisons were performed between the two groups and between the left and right sides for the EMG parameters.

**Results:**

No significant group or side differences between the slopes of the different measures used were found at the level of the apex (around T10) of the major curve of the spine. However, a significant side difference was seen at a lower level (L3, p = 0.01) for the MF/time parameter.

**Conclusion:**

The EMG parameters used in this study could not discriminate between the back muscles of scoliotic subjects and those of control subject regarding fiber type composition, neuromuscular efficiency and muscle fatigue at the level of the apex. The results of this pilot study indicate that compensatory strategies are potentially seen at lower level of the spine with these EMG parameters.

## Background

Scoliosis can be biomechanically described as a three-dimensional deformity of the spine, with deviations from the physiologic curves in the sagittal and frontal planes, usually combined with intervertebral rotation [1]. The disease often occurs during childhood or adolescence. It can be associated with congenital malformation of one or many vertebraes, fracture and/or dislocation of the spine, leg length discrepancy, hormone imbalance, habitual poor posture or by pain and muscle spasms [2]. When the deformity cannot be associated with any of the aforementioned causes, it is then labelled as "idiopathic scoliosis". Idiopathic scoliosis is the most common diagnosis given to a deviation of the spine [2,3] and despite the fact that a considerable number of studies aimed at explaining its etiology, the cause of idiopathic scoliosis is still unknown.

### Back muscle composition and idiopathic scoliosis

Biopsy studies shows abnormalities in the paraspinal muscles of scoliotic subjects concerning their architecture [4], their protein synthesis [5] and their muscle fiber type composition [1,6,7]. Even if many authors have investigated the alteration of the muscle fiber characteristics of paraspinal muscles with regards to idiopathic scoliosis, it is still not known if the observed differences are the cause of the disease or a consequence of it [1,6,7]. Idiopathic scoliosis affects mostly young women [8] and the major curve is often located in the thoracic spine. These facts raise a difficulty when one tries to explain the etiology of this disease through the alteration of muscle fibers because there is not much information on healthy backs, especially with regards to women and to muscles of the thoracic spine. Sirca & Kostevc [7] found that there was more type I than type II muscle fibers in the thoracic spine. However, their study included male subjects only. Mannion et al. [6,9] have investigated the muscle fiber composition in both genders at T10 and L3 levels. In their female subjects group, type I muscle fibers were present in a larger proportion than type II muscle fibers at the level of the thoracic spine. Moreover, type I muscle fibers presented a larger diameter at the thoracic level than at the lumbar level and also had a larger diameter than type II muscle fibers at both levels of the spine.

When one looks at the muscle fiber composition of scoliotic subjects, an important factor is whether or not the muscles are located on the concave or the convex side of the major spinal curve. Studies have demonstrated that the concavity of the curve shows alteration of fiber characteristics [6,10,11]. In general, in the muscle of scoliotic subjects taken at the same level of the spine (apex around T9, T10), the proportion of type I muscle fibers is smaller on the concave side than on the convex side whereas in normal subjects, a symmetric distribution is observed. However, type I muscle fibers are still more numerous than type II muscle fibers on both sides of the apex [6,10]. The other important difference is that the cross sectional area (CSA) of type II muscle fibers is larger on the concave side than on the convex side. Moreover, this CSA of the type II fibers is larger than in normal subjects [6,12]. Spencer and Eccles [11] have proposed that the difference on the concave side is due to a disparity in the proportion of each fiber type (type I fibers in a smaller percentage) rather than a difference in their relative size.

Surface EMG has been used as a non-invasive correlate to different muscle intrinsic properties such as muscle fiber composition, neuromuscular efficiency (related to weakness) and muscle fatigue. Obviously, the detection of differences in muscle fiber composition would be the primary focus of an EMG study. However, it would also be of interest to determine if scoliotic subjects also show other muscle impairments such as muscle weakness and fatigue. The rationale that justify the use of surface EMG to measure each of these muscle intrinsic property will be developed in the next sections.

### Assessment of muscle composition with the EMG MF/force relationship

Muscle biopsy remains the technique commonly used to study muscle fiber type composition. However it has been suggested that the MF/force relationship could potentially be used as a non-invasive measure of muscle fiber composition and muscle fiber size [13,14]. The conduction velocity of the muscle fibers has been shown to be proportional to the diameter of the recruited muscle fibers [15]. The central tendency statistics such as the MF and the mean power frequency (MPF) of the EMG power spectrum are highly correlated with the average muscle fiber conduction velocity [13,16,17]. Thus, it is expected that the values of the MF or the MPF will increase with the increasing force level due to the recruitment of larger type II muscle fibers [14,18,19]. However, with atypical muscles such as the erector spinae where the predominant type I fibres have an equal or larger diameter than type II fibres [9,20], the MF remains usually stable or decreases in some cases across the force levels [21].

### Assessment of neuromuscular efficiency with the EMG RMS/force relationship

One EMG measure of interest associated with the functional capacity of a muscle is linked to the "neuromuscular efficiency" concept [22]. This concept suggests that a weak subject would produce more EMG than a stronger subject to generate a given absolute force, thus less efficient muscle contractions are characterised by steeper RMS/force relationship slopes [23,24]. This concept has been used to study muscles of subjects suffering from neck pain [25] and cerebro-vascular accident (CVA) [26]. It appears that with reliable EMG parameters, some differences in efficiency are observed among subjects who are experiencing pain [25] whereas among CVA subjects, it was only partially demonstrated [26].

### Assessment of muscle fatigue with the EMG RMS/time and MF/time relationships

To sustain a sub-maximal contraction for a substantial amount time, the recruitment of new motor units is necessary. While the contraction is ongoing, the muscle produces more EMG [27]. When the EMG amplitude (RMS) is plotted against time, the slope of the RMS/time relationship is generally positive. However, when one looks at the median frequency (MF) of the power spectrum over time, the MF/time slope presents a negative relationship demonstrating a shift toward lower frequencies. The MF decrease of the EMG power spectrum during a fatiguing muscle contraction is mainly attributed to the decrease of the conduction velocity [28], which reflects the accumulation of metabolic products on the surface area of the muscle fibers. That could also explain the shift of the MF of the power spectrum towards lower frequencies. The EMG RMS and MF of the power spectrum can thus be considered sensitive measures of muscle fatigue [27,29].

The above EMG measures have been successfully used to study the paravertebral muscle activity in normal and back pain subjects [21,30-32]. Before using a similar measurement protocol in a cohort of scoliotic subjects and considering the difficulty of recruiting scoliotic patients before surgery, a pilot study was conducted on a small number of patients. The main objective of this pilot study was to examine the clinical potential of these different EMG parameters for assessing the back muscles of patients presenting idiopathic scoliosis, using a triaxial dynamometer allowing the measurement of asymmetric efforts during extension contractions. More specifically, the sensitivity of these EMG measures (amplitude and MF) to detect muscle weakness and muscle fatigue was assessed among scoliotic subjects and contrasted with normal subjects. Also, the sensitivity of these EMG parameters to the known muscle morphological differences between the concave and convex side in scoliotic subjects was explored.

## Methods

### Subjects and tasks

The control group (CG) was composed of ten young women with no back problem or physical disability. They were recruited among the children of parents attending a physiotherapy clinic. Subjects were excluded if they had experienced back pain in the last six months prior to the experiment. Six young women with a diagnosis of idiopathic scoliosis formed the scoliotic group (SG) and they were recruited from the Clinique de la Scoliose of Ste-Justine Hospital in Montreal. Any scoliotic subject having experienced episodes of back pain in the last six months prior to the experiment, who was on a specific treatment for the scoliosis, had been wearing a corset for more than three months at the time of the experiment or for more than six months prior to the experiment was excluded. Explanations concerning the experimental protocol were given and a written consent form was signed by all the subjects and their parents. The subjects' characteristics were for the scoliotic group (age: 16 ± 3.5 years; height: 160.6 cm ± 10.2; mass: 51.3 Kg ± 8.5; L5/S1 peak extension moment: 100.2 Nm ± 40.2; handedness: all right; Cobb's angle (all right thoracic curve, apex T8–T10): 35.6° ± 4.2) and for the control group (age: 14.4 ± 2.6 years; height: 154.6 cm ± 10.4; mass 44.8 Kg ± 8; L5/S1 peak extension moment: 100.7 Nm ± 33.3; handedness: all right).

After the EMG electrodes were positioned and the subject was stabilized in the static dynamometer, two to three submaximal extension contractions were performed in order to get used to the apparatus. Then, two maximal voluntary contractions (MVC) in extension were performed and the highest value was kept to obtain a reference value for the following tests. The neuromuscular efficiency test consisted of three 7 s ramp extension contractions from 0% to 100% MVC separated by a two minute rest period. The rate of the contraction had to be paced with the speed of a target. If this condition was not respected, the trial was rejected and repeated after an adequate rest period. The ramp demonstrating the best control was kept for data processing.

For the muscle fatigue test, the subjects performed a 30 s extension static contraction at 75% MVC as proposed by van Dieen & Heijblom [33]. Again, visual feedback was given to the subject on a computer screen located in front of her.

### Dynamometry

**Figure 1:**
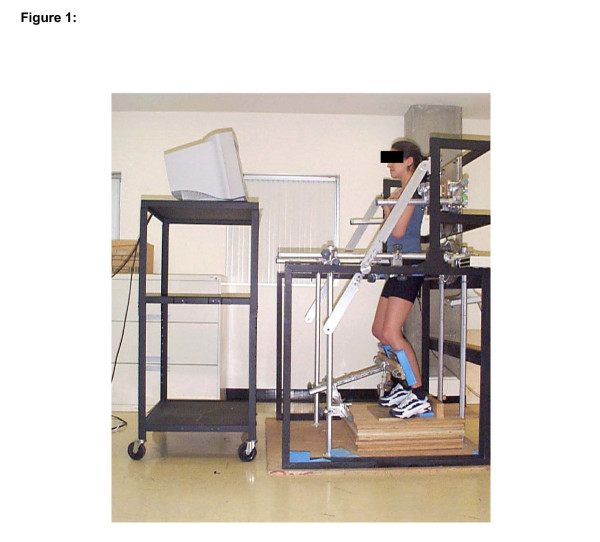
Subject positioned in the triaxial dynamometer with the sign convention of the AMTI force platform. Positive My = extension moment, positive Mx = right lateral bending moment and positive Mz = left axial rotation moment.

The subjects stood in a dynamometer which consisted of a triaxial force platform (Advanced Mechanical Technology Inc., model MC6-6-1000) allowing simultaneous measurements of static moment generated around the trunk three axes (flexion-extension, lateral flexion and rotation) at the level of L5-S1 joint during an isometric effort in back extension [34]. The force platform was fixed on a metal frame and could be adjusted at the superior part of the trunk to be positioned at the level of T4. The pelvis, the knees and the feet were stabilized to minimize movement of the lower body and to isolate back muscle contractions (Figure 1). The knees were kept in a slightly flexed position. The L5/S1 extension moments were computed in real time and provided to the subjects as visual feedback on a monitor positioned in front of them. The visual feedback consisted of a vertically moving target with lower and upper bounds corresponding to a tolerance limit of ± 10% MVC. It was used to control the pace of the contraction rate for the neuromuscular efficiency test and to make sure that the isometric contraction of the fatigue test was as steady as possible.

### Electromyography

Eight active surface electrodes (Model DE-2.3, Delsys Inc., Wellesley, MA) were used to collect the EMG signal. Each pair of electrodes is composed of two silver bars 10 mm long and 1 mm wide encastrated in non-conductive material and separated from each other by 10 mm. The EMG signal was bandpass filtered (20 – 450 Hz), pre-amplified with a gain of 1000, analogue to digital converted at a sampling rate of 4096 Hz and stored on a hard disc for later analysis. A custom Lab View software (National Inst.) was used to collect and process force and EMG data.

Electrode sites were identified (details below) and the thickness of subcutaneous tissues at these electrode sites was measured twice on each side with a Harpenden skinfold caliper. Afterwards, the skin at the electrode sites was abraded and cleaned with alcohol. The electrodes were positioned on each side of the following muscles respecting the muscle fibers direction [35]: Multifidus at L5, Iliocostalis at L3, Longissimus at L1 and T10. One ground electrode (snap-on type) was positioned on the spinous process of T8.

### Data processing and statistical analyses

For the neuromuscular efficiency test, 250 ms windows were selected from the EMG signals at each of the following force levels: 10, 20, 30, 40, 50, 60, 70, 80% MVC. The signal within each window was transformed in RMS and in MF of the EMG power spectrum (fast Fourier transform, Hanning window processing). A spectral resolution of 4 Hz was then possible with respect to MF for the efficiency test (1024 points in a 250 ms window and with a sampling frequency of 4096 Hz). The RMS/force and MF/force relationships of each muscle were determined using linear regression. A linear RMS to force relationship was assumed in the present study based on other studies showing either a linear [36,37] or a quasi linear [37] relationship depending on the selected back muscle.

The slope values for the RMS/force relationship were indicators of neuromuscular efficiency, thus weaker muscles were characterized by steeper slopes. The slope values for the MF/force relationship reflected the average conduction velocity of the muscle and indirectly, muscle fiber type composition.

For the fatigue test, both RMS and MF values were calculated on a succession of 21 windows (250 ms) equally placed from the 3^rd ^second to the 25^th ^second of a contraction lasting 30 seconds at 75 % MVC. The RMS/time and the MF/time relations were also studied using linear regression techniques and the slope values were indicators of muscular fatigue.

To verify if scoliotic subjects produced more asymmetric efforts than controls during the neuromuscular efficiency and fatigue tests, the coupled (lateral bending, axial rotation) L5/S1 moments were computed at the same time-windows as for EMG analyses. For each series of data (efficiency test: 8 values; fatigue test: 21 values) and each subject, the mean, the minimum and the maximum values were determined. Student unpaired t-tests were used to compare groups characteristics such as age, height, body mass, skinfold thickness at each electrode site and to compare L5/S1 peak extension (during MVC tasks) and coupled moments (during the efficiency and fatigue tasks).

Two-way ANOVAS (2 Groups × 2 Sides) with one repeated measure (side) were used to compare the slope values between the two groups and between left and right sides for the EMG parameters of both the efficiency and the fatigue tests. The level of statistical significance was set at 0.05.

## Results

### Description of subject samples

No intergroup differences were observed by the Student t-tests performed for age (p = 0.36), height (p = 0.28) and body mass (p = 0.16). The peak L5/S1 moment produced in extension was equivalent for both groups as disclosed by a Student's t-test (p = 0.98).

Concerning the skinfold thickness, although the subjects of the scoliotic group tended to present higher values for all muscle sites, no significant difference was shown when these values were compared with those of the control group. Similarly, regarding the side factor, again no difference was observed within each group (paired Student t-test p > 0.05).

During the efficiency tasks, the coupled L5/S1 moments in lateral bending and axial rotation were not significantly different between groups (t-test, p > 0.05). There were no significant intergroup differences either for mean, the minimum and the maximum values in both L5/S1 coupled moments (t-test, p > 0.05). In lateral bending (positive values = right lateral bending), the mean, the minimum and the maximum values were respectively 0.85, -0.56 and 2.5 Nm for the control subjects and 0.34, -3.27 and 1.81 Nm for the scoliotic subjects. In axial rotation (positive values = left axial rotation), the mean, the minimum and the maximum values were respectively 1.86, -0.33 and 4.28 Nm for the control subjects and 1.97, -1.53 and 5.34 Nm for the scoliotic subjects.

Similar results were obtained during the fatigue test as no group differences (Student's t-test, p > 0.05) were observed. In lateral bending the mean, the minimum and the maximum values were respectively 1,62, -3,57 and 6,62 Nm for the control subjects and -1.28, -4.37 and 0.71 Nm for the scoliotic subjects. In axial rotation, the mean, the minimum and the maximum values were respectively -2.56, -5.36 and 0.42 Nm for the control subjects and -1.50, -2.84 and 0.82 Nm for the scoliotic subjects.

### Assessment of muscle composition

No significant difference in the MF/force relationships between the two groups were observed for all eight muscles investigated. Regarding side differences, the iliocostalis (L3) showed a nearly significant difference (p = 0.09) (Table 2). The MF/force slopes were negative for the right and left multifidus muscles (L5) as well as for the right iliocostalis muscle (L3) in the normal group. In the scoliotic group, the slopes were negative on both sides of the multifidus (L5) and iliocostalis (L3) as well as on the right side of the longissimus (L1).

**Table 2:** Summary of the two way ANOVAs with one repeated measure on the side factor for each muscle investigated for the RMS/force, MF/force, RMS/time and MF/time relationships

		RMS/Force		MF/Force		RMS/Time		MF/Time	
		F	p	F	p	F	p	F	p
	
L5	Group	1.00	0.39	0.66	0.53	0.04	0.96	0.50	0.62
	Side	1.78	0.20	0.21	0.65	0.04	0.84	0.21	0.65
	Group × side	0.63	0.44	0.82	0.38	0.02	0.89	0.56	0.47
	
L3	Group	1.72	0.22	1.61	0.23	0.46	0.62	4.59	0.17
	Side	2.52	0.14	3.13	0.09	0.00	0.97	8.28	0.01*
	Group × side	1.75	0.21	0.02	0.89	0.87	0.37	2.69	0.12
	
L1	Group	0.14	0.87	0.20	0.82	0.34	0.72	0.56	0.59
	Side	0.10	0.76	0.41	0.53	0.02	0.88	0.18	0.68
	Group × side	0.10	0.75	0.01	0.91	0.56	0.47	1.08	0.32
	
T10	Group	0.71	0.51	0.16	0.85	0.97	0.40	0.09	0.92
	Side	1.15	0.30	0.11	0.75	0.11	0.75	0.13	0.73
		0.61	0.45	0.12	0.73	1.95	0.18	0.09	0.77

* : Statistically significant

### Assessment of neuromuscular efficiency

Table 1 presents the mean values of the slopes of the regression line for each measure used for a given muscle for both the control group and the scoliotic group. Table 2 presents the summary of the two-way ANOVAs with one repeated measure for each of the muscle investigated and each of the measures used.

**Table 1:** Descriptive statistics for each measure used (slope of a regression line) for a given muscle for both the control (CG, n = 10) and the scoliotic group (SG, n = 6)

Mucles	Groups		Slope value measures (mean (SD))		
		RMS/Force	MF/Force	RMS/Time	MF/Time
					
L5 (L)	SC	0.80 (0.50)	-0.18 (0.38)	0.60 (0.46)	-1.04 (0.53)
	CG	0.98 (0.53)	-0.24 (0.41)	0.54 (0.67)	-0.75 (0.82)
					
L5 (R)	SC	0.81 (0.48)	-0.21 (0.26)	0.83 (0.86)	-0.92 (0.92)
	CG	0.99 (0,55)	-0.08 (0.59)	0.59 (0.68)	-1.12 (0.73)
					

L3 (L)	SC	0.83 (0.56)	-0.03 (0.33)	0.54 (0.41)	-0.31 (0.64)
	CG	1.29 (0.82)	0.00 (0.17)	0.84 (1.28)	-0.28 (0.51)
					
L3 (R)	SC	0.67 (0.43)	-0.41 (0.24)	1.22 (1.17)	-0.70 (0.64)
	CG	1.44 (1.42)	-0.10 (0.27)	0.90 (1.08)	-0.60 (0.57)
					

L1 (L)	SC	1.32 (0.81)	0.07 (0.31)	0.63 (0.72)	-0.52 (0.37)
	CG	1.08 (0.46)	0.08 (0.19)	0.72 (1.01)	-0.57 (0.60)
					
L1 (R)	SC	1.26 (1.14)	-0.02 (0.22)	0.63 (0.46)	-0.38 (0.37)
	CG	1.18 (0.66)	0.11 (0.18)	0.63 (0.74)	-0.48 (0.45)
					

T10 (L)	SC	0.68 (0.47)	0.10 (0.24)	0.86 (0.56)	-0.49 (0.19)
	CG	0.87 (0.37)	0.16 (0.33)	0.58 (0.97)	-0.33 (0.38)
T10 (R)	SC	0.79 (0.64)	0.04 (0.30)	0.44 (0.39)	-0.49 (0.48)
	CG	0.80 (0.41)	0.15 (0.22)	0.51 (0.92)	-0.23 (0.63)

R/L: Right/Left; L5: Multifidus muscle; L3: Iliocostalis muscle; L1: Longissimus muscle; T10: Longissimus muscle

For the EMG signals, no significant differences were found in the RMS/force relationships obtained between the scoliotic and control groups or between the left and right sides. This was true for each muscle pair investigated (Table 2).

### Assessment of muscle fatigue

As expected, the slopes of the RMS/time relationships were all positive for all muscles in both groups (Table 1). There were no significant differences in the RMS/time relationships (fatigue test) between groups and between sides for each muscle pair (Table 2).

**Figure 2:**
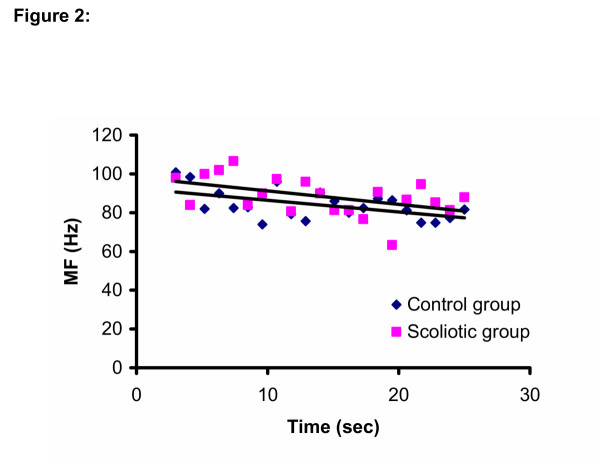
MF/time relationship for both groups for the left Iliocostalis muscle at the level of L3.

**Figure 3:**
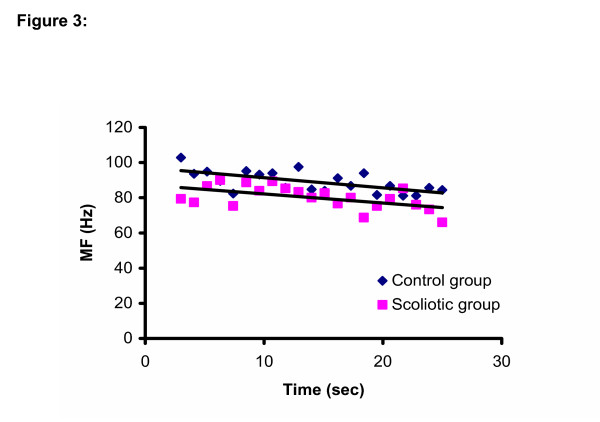
MF/time relationship for both groups for the right Iliocostalis muscle at the level of L3.

For the MF/time relationships, there was a side difference (p = 0.01) for the iliocostalis (L3) muscles, the right side showing more negative slopes (more fatigue) than the left side. However the Group × Side interaction was not significant (Table 2). The slopes were all negative for all muscles in both groups (Figure 2 and Figure 3)

## Discussion

### Control of potential confounders

One factor of importance when interpreting EMG signals is the skinfold thickness, since it may act as a biological filter [38]. Fortunately, no differences were found between subjects, groups and sides. However, the small number of subjects cannot fully support the control of this confounding variable and this will be taken into account in future research protocols developed from those preliminary data.

Before contrasting the EMG results of the control and scoliotic subjects, it was also important to verify if the efforts performed in the different planes were similar. Although it cannot be ascertained that the back muscle forces were the same between groups, the analysis of the principal (extension) and coupled (lateral bending and axial rotation) L5/S1 moments demonstrated that the main efforts were comparable. Thus, contrary to what might be expected from their asymmetric deformity of the spine, scoliotic subjects performed coupled L5/S1 moments comparable to those of the control subjects. This is a new finding in this literature. Furthermore, the small coupled moment values, which were either within or close to the maximal measurement errors of the dynamometer (lateral bending: 1 Nm; axial rotation: 8.8 Nm) [34], were negligible from a physiological point of view.

### Assessment of muscle composition

As seen in the introduction section, there is an alteration of the muscle fiber type composition on the concave side of the thoracic curve in scoliotic subjects. The percentage of type I muscle fibers is reduced in the paraspinal muscles on the concave side relative to the convex side and relative to the homologous muscle of non scoliotic subjects [6,10]. The cross sectional area occupied by type II muscle fibers is larger in the paraspinal muscles on the concave side than in those on the convex side and is also larger than in the paraspinal muscles of normal subjects [6]. The average muscle conduction velocity should be faster in the muscles on the concave side than in those on the convex side. Considering this, a more pronounced increase of the conduction velocity, and consequently, of MF values, would have been expected in the paraspinal muscles on the concave side of the thoracic curve of scoliotic subjects as the force level increases. This was shown in other studies using the muscles of the extremities [23,27,28,39]. However, the interpretation of the relationship between muscle fiber composition (proportions, areas) and spectral parameters is non-conclusive in the literature [22,40-42] and this section of the study was done on an exploratory basis. The MF/force slope showed no side difference in the present study at the level of the thoracic spine. It is possible that our scoliotic subjects were not affected enough (small Cobb angle, the younger subjects may not have altered muscle fiber type composition yet) by their condition in order to see major alterations in the EMG signals. The lack of difference can also demonstrate that either this measure was not sensitive to the back muscle composition of this population or that there was no difference in the back muscle composition of the subjects evaluated.

It is of interest to note that for the muscles at the level of L5, the values of the MF were compressed towards the lower frequencies of the spectrum as the force level was increasing. This could be explained by the recruitment of type II muscle fibers of the multifidus muscle which are known to be smaller than type I muscle fibers [29].

### Assessment of neuromuscular efficiency

The peak L5/S1 extension moment showed no group difference. This concurs with other comparative studies contrasting scoliotic and normal subjects [43,44]. Given the same net moment and that the effort was symmetric, we could speculate that the EMG would be the same in both groups and on both sides. This was demonstrated with the results of the present study, where no significant differences were found in the RMS/force relationships obtained between the scoliotic and control groups or between the left and right sides and this, for each muscle pair investigated.

Contrary to the present findings, data from other studies demonstrated that muscles of the back on the convex side of the curve of scoliotic subjects produced more EMG than homologous muscles of healthy subjects and that they also produced more EMG than muscles on the concave side [45-47]. However, it cannot be verified from the data of previous studies if the back asymmetric efforts in extension, which could have produced these EMG differences, were similar between groups. Nevertheless, these differences can also be associated with different experimental procedures involving different position of the subject as well as different tasks performed.

### Assessment of muscle fatigue

The RMS/time slopes were all positive indicating that the muscles generated more EMG to maintain the level of the contraction for the required time to perform the task. However, no group or side differences were disclosed (Table 2). Likewise, as seen in previous studies on scoliotic subjects [46] and low back pain subjects [32,48], the slopes of the MF/time relationship were all negative, also indicating the presence of muscle fatigue. In the present study, no between-group differences were shown for this parameter. Thus, since the L5/S1 peak extension moment and the MF/time relationship were equivalent in both groups, the muscle endurance was similar and this for all the muscles evaluated. This is in accordance with the results of Zetterberg [46], although it is not mentioned if their subjects have performed similar symmetric efforts.

The MF/time slope showed a significant side effect in the iliocostalis muscle at the level of L3 but the Group × Side interaction was not significant. A side difference at the lumbar spine was shown in another study on muscles of low back pain subjects [32] and one possible explanation can be related to side dominance [45]. The back muscles on the non-dominant side showed less muscle fatigue and this was significant in the right handed population [49]. This is in accordance with the results of the present study where all subjects were right handed and where the MF/time slopes on the right side were more negative, indicating greater signs of muscle fatigue.

Some limitations of the study must be addressed. Group or side discrimination in the muscle efficiency and in the muscle fatigue tasks may not have been possible in the muscles of the thoracic spine due to the small number of subjects. Data obtained from the present study will be used to carry out some power calculations in order to determine the number of subjects needed to demonstrate a clinically significant difference in the main outcome measures between scoliotic subjects and controls in the next step of this research. It is also a possibility that the scoliotic subjects who participated to the study were not impaired enough to show any alteration in their muscle function and or in their muscle fibers.

## Conclusion

The EMG parameters used in this study could not discriminate the back muscles of scoliotic subjects from those of control subject regarding muscle fiber composition, neuromuscular efficiency and muscle fatigue at the level of the apex, where abnormal muscle fiber composition is observed from biopsy data. However, the results of this pilot study showed a side difference for muscles located at lower level of the spine (L3). That might be an indicator that the erector spinae at the thoracic level might rely on compensatory strategies involving muscles located in the lumbar area to maintain a specific torque level during a fatigue task.

## Competing interests

The author(s) declare that they have no competing interests.

## Authors' contributions

NG is a physical therapist and a Ph.D. candidate in the Biomedical Sciences program at University of Montreal. This article is derived from her master thesis. She was involved in all the research processes. AB is the director of the School of Rehabilitation at University of Montreal, Montreal, Québec, Canada. As the research director of Mrs Gaudreault, he was also involved in all the steps of the project, mainly in the conception and the design of the research protocol. CL is a researcher at l'Institut de recherche Robert-Sauvé en santé et sécurité au travail (IRSST), Montréal, Québec. From its expertise in the use of the EMG signal, he was mainly involved during the data acquisition, analysis and interpretation of the results. He also contributed in drafting the article and revising it critically. SD is a professor at the School of Physical and Occupational therapy at McGill University. Her help was precious during the design of the protocol and for the interpretation of the data. She contributed through her comments to the manuscript. CHR is an orthopeadic surgeon working with scoliotic children at Ste-Justine hospital, Montreal, Québec, Canada. He contributed to the project as the medical expert and took part of the recruitment process. All authors read and approved the final manuscript.

## Pre-publication history

The pre-publication history for this paper can be accessed here:


